# A new genus of Pelecotominae from Mexico, with notes on the genera *Clinops* and *Scotoscopus* and the description of new species (Coleoptera, Ripiphoridae)

**DOI:** 10.3897/zookeys.857.34938

**Published:** 2019-06-25

**Authors:** Michael S. Engel, Zachary H. Falin, Jan Batelka

**Affiliations:** 1 Division of Entomology, Natural History Museum, 1501 Crestline Drive – Suite 140, University of Kansas, Lawrence, Kansas 66045-4415, USA; 2 Department of Ecology & Evolutionary Biology, University of Kansas, Lawrence, Kansas 66045, USA; 3 Division of Invertebrate Zoology, American Museum of Natural History, Central Park West at 79th Street, New York, New York 10025-5192, USA; 4 Department of Zoology, Faculty of Science, Charles University, Viničná 7, 128 43 Praha 2, Czech Republic

**Keywords:** distribution, Greece, Mexico, South Africa, taxonomy, Tenebrionoidea

## Abstract

Taxonomic notes are provided on species of the uncommonly encountered ripiphorid subfamily Pelecotominae. *Zapotecotomasumichrasti***gen. et sp. nov.**, is described from southern Mexico based on a unique male likely collected in the later part of the mid-19^th^ Century. The discovery of additional species of the South African genus *Clinops* Gerstaecker permit a revised diagnosis and distinction of the group from the eastern Mediterranean genus *Scotoscopus* Brenske and Reitter, resurrected status. Two new species of *Clinops* are established: *Clinopsinexpectatus***sp. nov.** (northeast of Durban near Swaziland) and *C.perpessus***sp. nov.** (region of Durban), and *Scotoscopusspectabilis* (Schaufuss) is newly recorded for the Peloponnese in Greece.

## Introduction

The ripiphorid subfamily Pelecotominae is one of the earliest diverging lineages of wedge-shaped beetles, only the Ptilophorinae being more basal in the phylogeny of Ripiphoridae (Falin 2003; Batelka et al. 2016a). Pelecotominae are infrequently encountered beetles with most of their scant diversity (hitherto 14 modern species in eight genera: table 1) found in the New World tropics, and other species scattered from eastern North America, central and southern Europe, Turkey, South Africa, Japan, mainland Malaysia, and New Zealand (Falin 2003; Batelka 2009). Where known, species are parasitoids of the larvae of wood-boring beetles (Ptinidae [formerly Anobiidae] and Cerambycidae) (Hudson 1934; Watt 1983; Kuschel 1990; Švácha 1994; Batelka 2005; Lawrence et al. 2010). Not surprising given its phylogenetic position, Pelecotominae are known from as far back as the mid-Cretaceous, and from a diversity of species largely preserved in amber from northern Myanmar (Batelka et al. 2016b, 2018; Hsiao and Huang 2018). All other Cretaceous species of Ripiphoridae belong to the more derived subfamily Ripidiinae (e.g., Falin and Engel 2010; Batelka et al. 2016b, 2018; Cai et al. 2018).

The subfamily has been generally characterized by Falin (2003) who incorporated therein the former Micholaeminae which otherwise rendered the more traditionally restricted Pelecotominae paraphyletic. Inclusive of the micholaemines, the subfamily can be distinguished from other Ripiphoridae by the long and slender body form with fully developed elytra, protibiae shorter than the protarsi, labrum connected to the frontoclypeus, presence of a small dorso-ventral phragma on the inner aspect of the anterior edge of the mesepisternum, and a U-shaped excavation on the ventral margin of the fossa of the pronotum (the latter two serving as synapomorphies for the clade according to Falin 2003). The genera are poorly understood owing to a dearth of available material and are generally characterized by an intermingling of features relating to the tibial spur formula, form of the pretarsal claws, and form of the maxillary palpomeres. Although some degree of relationship was recovered in the morphological analyses of Falin (2003), considerable phylogenetic work remains to be undertaken, particularly once additional material is discovered, proper dissections undertaken, and ideally both sexes for the various genera characterized (presently, the majority of species are known only from one sex).

Herein we provide various notes on Pelecotominae. First, we describe a new genus and species from southern Mexico, thereby allowing us to update the tabulation of known diversity within the subfamily (Table 1). In addition, we clarify the diagnosis of the genus *Clinops* Gerstaecker, and describe two new species of this southern African genus. In clarifying the identity of *Clinops*, we resurrect the genus *Scotoscopus* Brenske and Reitter for a species known to occur in Greece and Turkey.

**Table 1.** Currently recognized genera of Pelecotominae (sensu Lawrence et al. 2010). Daggers (†) denote extinct taxa.

**Genus**	**No. Species**	**Spur Formula**	**Distribution**
*Allocinops* Broun, 1921	1	1-2-2	New Zealand
*Ancholaemus* Gerstaecker, 1855a	2	0-1-2	Brazil, Ecuador (Galapagos Islands)
†*Burmitoma* Batelka, Engel, & Prokop, 2018	1	0-2-2	Myanmar (Cenomanian)
*Clinopalpus* Batelka, 2009	1	0-0-1	mainland Malaysia
*Clinops* Gerstaecker, 1855a	3	0-0-2	South Africa
†*Flabellotoma* Batelka, Prokop, & Engel, 2016b	1	0-0-0	Myanmar (Cenomanian)
*Micholaemus* Viana, 1971	1	0-1-2	Argentina
*Pelecotoma* Fischer von Waldheim, 1809	3	1-1-1	eastern North America, central Europe, Japan
†*Plesiotoma* Batelka, Engel, & Prokop, 2018	1	1-2-2	Myanmar (Cenomanian)
*Rhipistena* Sharp, 1878	3	2-2-2	New Zealand
*Scotoscopus* Brenske & Reitter, 1884	1	0-2-2	Greece, Turkey
*Sharpides* Kirkaldy, 1910	1	2-2-2	New Zealand
†*Spinotoma* Hsiao & Huang, 2018	1	?	Myanmar (Cenomanian)
*Zapotecotoma* gen. nov.	1	0-1-1	southern Mexico

## Materials and methods

Morphological terminology and the format for descriptions generally follow that used elsewhere for Pelecotominae (e.g., Falin 2003; Batelka 2005, 2009; Batelka et al. 2016b) and more generally for Ripiphoridae (e.g., Lawrence et al. 2010). In reporting specimen label data we have separated information on separate lines of a single label with single slashes (/), and material on separate labels with double slashes (//). Annotations of added information meant to clarify otherwise ambiguous abbreviations or provide further insight into label data is provided in brackets. Material recorded herein is deposited in the following institutions:

**MCZ**Museum of Comparative Zoology, Harvard University, Cambridge, Massachusetts, USA (P. Perkins).

**NHMUK**The Natural History Museum, London, United Kingdom (M. Barclay).

**TMSA**Ditsong National Museum of Natural History (formerly the Transvaal Museum), Pretoria, South Africa (R. Müller).

**ZMUC**Statens Naturhistoriske Museum, Universitetets Zoologiske Museum, Copenhagen, Denmark (A. Solodovnikov).

## Systematics

### Family Ripiphoridae Laporte

#### Subfamily Pelecotominae Seidlitz

##### Zapotecotoma gen. nov.

Taxon classificationAnimaliaColeopteraRipiphoridae

http://zoobank.org/958B329B-973A-4D01-9FC7-0DC1E3581C82

###### Type species.

*Zapotecotomasumichrasti* sp. nov.

###### Diagnosis.

♂: Body slender; head with postocular genae expanded into lobes; compound eye not expanded beyond mandibular base and with a small extra-antennal sclerotous emargination; antenna with eleven antennomeres; antennomeres I–III simple, IV–X with inner-facing, flabellate, compressed rami, XI similar in shape to preceding rami; ultimate maxillary palpomere cylindrical, not compressed or expanded; distal sensory duct on ultimate maxillary palpomere a small, ovoid point. Lateral aspect of pronotum with a ventrally bowed sulcus; pronotal disc without longitudinal medial impression; mesosternum convex but without distinct medial keel; metepisternum with elytron-receiving carina extending along anterior portion only; posterior aspect of metepimeron narrow. Metacoxa with strongly developed posterior flange; ventral surface of pro- and mesofemora in males without densely setose patch; tibial spur formula 0-1-1; pretarsal claws bifid.

♀: Unknown.

###### Etymology.

The new genus-group name is a combination of Zapotec, the principal indigenous people in the region of the type locality, and –*toma* (derived from the Greek, *tome* or *tomeus*, meaning, “separation”, “cutting”, or “cutter”), a suffix generally used in the generic names of pelecotomines. The gender of the name is feminine.

##### Zapotecotoma sumichrasti sp. nov.

Taxon classificationAnimaliaColeopteraRipiphoridae

http://zoobank.org/DCF375E0-9DC9-4AF4-9F39-B72F1630D911

Figs 1–2 , 3

Pelecotominae new genus 1 gen. nov.: Falin 2003: 184.

###### Diagnosis.

As for the genus (*vide supra*).

**Figures 1, 2.**
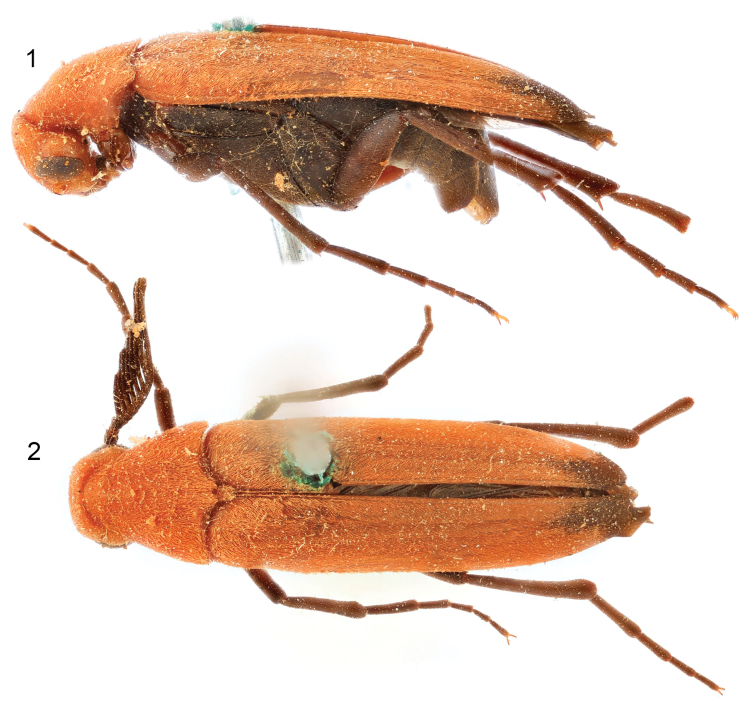
*Zapotecotomasumichrasti* gen. et sp. nov., holotype male. **1** lateral habitus **2** dorsal habitus.

**Figures 3–5.**
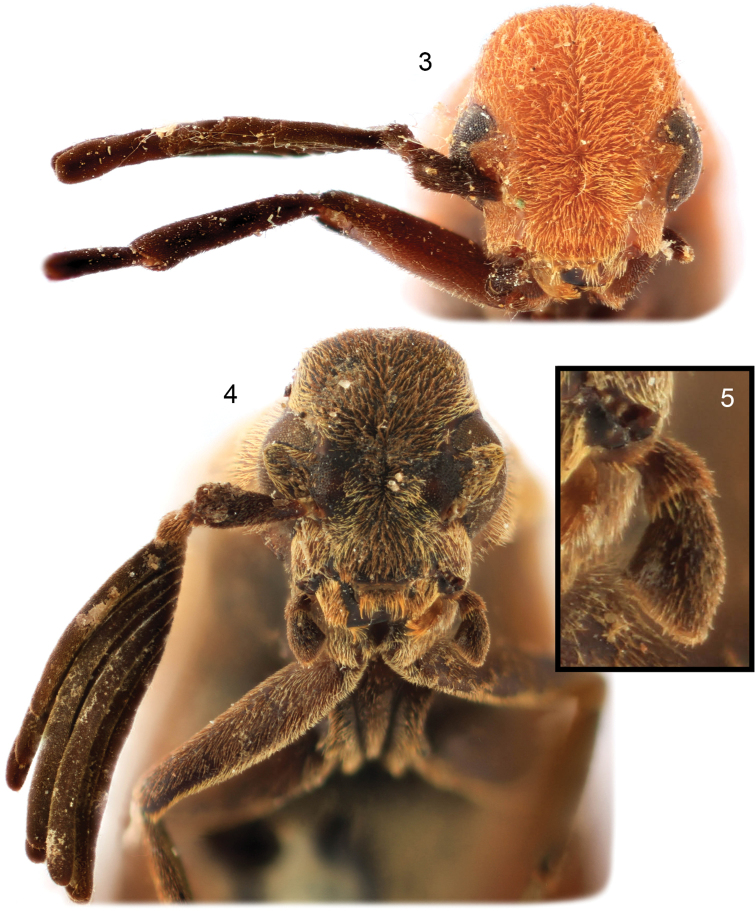
Details of pelecotomine genera. **3** facial view of *Zapotecotomasumichrasti* gen. et sp. nov., holotype male **4** facial view of *Clinopsperpessus* sp. nov., holotype male **5** inset detail of maxillary palpus of *C.perpessus*.

###### Description.

♂: General size and appearance typical of Pelecotominae. Size 7.38 mm from tip of abdomen to base of antennae, 2.15 mm wide at base of pronotum. Body bicolorous; head, prothorax, mesoscutellum, and majority of elytra orange testaceous; remainder of body dull, dark reddish brown, including patches at apexes of elytra (Figs 1–2).

Head ovoid, approximately 1.1× longer than wide in facial view, medial length 1.67 mm, maximum width (across compound eyes) 1.54 mm. Vertex convex dorsally and posteriorly, as wide as lower face (below compound eyes), rising high above compound eyes in facial view, sloping uniformly to meet and slightly overlap pronotal anterior margin (Fig. 1), with weak medially impressed line, disappearing posteriorly. Dorsal, lateral, and facial aspects of head with fine, semi-decumbent, orange setae, particularly numerous on face between compound eyes and vertex, sparse on genae (Fig. 3); integument dull, with minute, weak, nearly contiguous punctures separated by apparently smooth or faintly imbricate integument (where evident). Compound eye small on middle third of lateral surface of head, finely faceted, emarginate in upper third. Postocular gena expanded into lobe. Frons broad, with antennal torulus laterally directed, antennal toruli separated by distance greater than length of scape, compound eyes separated by distance greater than compound eye length. Malar space elongate, more than one-half length of scape, slightly less than basal mandibular width. Mandible short, slightly curved, with short, acute subapical tooth. Maxillary palpus long, tetramerous, apical palpomere largest, cylindrical, its apical width approximately one third its maximum length, with acutely rounded apex, not flattened or grossly enlarged (greatly enlarged and flattened in *Ancholaemus* Gerstaecker and *Micholaemus* Viana), distal sensory duct point-like.

Antenna consisting of eleven antennomeres; antennomere I longer than wide, slightly curved to approximate compound eye; antennomere II short, slightly wider than long; antennomere III longer than antennomere II, about as long as apically wide, triangular, apical margin oblique so as to receive base of following antennomere. Antennomeres IV–XI greatly dissimilar from preceding antennomeres; antennomeres IV–X with internally facing, compressed rami; bases of antennomeres IV–X short and of similar lengths; rami IX and X elongate, extending to apex of antennomere XI (apexes of rami IV–VIII damaged and missing and left antenna completely missing so precise structure of all rami uncertain). Antennomere XI expanded, similar in shape to rami of preceding antennomeres. Total length of antennomere XI nearly 1.6× length of bases of antennomeres IV–X combined.

Pronotum with suberect to semi-decumbent, fine, orange setae, integument dull, and weakly, indistinctly, and contiguously punctate, with punctures more indistinct posteriorly and integument becoming imbricate. Pronotum triangular in shape, narrowed anteriorly; anterior margin broadly rounded; posterior margin sinuate and generally trilobed, with medial lobe as broad as mesoscutellum and narrowly emarginate, acutely rounded on either side of emargination; lateral margins generally straight, converging apically, convex ventrally to propleurae; propleuron well developed. Pronotal disc without mediolongitudinal carina or impression but with a weak transverse impression near apex and a pair of weak oblique impressions on either side of midline near base; lateral aspect with a ventrally bowed sulcus. Mesonotum obscured by elytra. Mesoscutellum short, flat, parallel-sided, with broadly rounded apex; integumental sculpturing and setation as on pronotal disc. Metanotum obscured by elytra.

Lateral and ventral aspects of pterothorax typical of subfamily. Mesepisternum weakly imbricate, fused with mesosternum, with scattered semi-decumbent setae. Mesepimeron forming prominent, rectangular sclerite separated from mesepisternum by deep sulcus; sculptured and setation as on mesepisternum. Metepisternum an elongate, narrow rectangular sclerite, with sculpturing and setation as on mesepisternum; metasternum massive, weakly imbricate and with semi-decumbent setae more numerous than on metepisternum. Metepimeron approximately parallel-sided except apically upper margin arching ventrally, extending anteriorly to wing base as narrow (slightly more narrow than metepisternum), sclerotized band; weakly imbricate with scattered fine setae.

Legs typical for subfamily; coxae, trochanters, and femora weakly, irregularly, almost indistinctly punctate on otherwise smooth and shining integument with semi-decumbent to suberect lightly fuscous setae; metacoxa with strongly developed posterior flange; femora without densely setose patches ventrally; tibiae straight, cylindrical, broadened slightly apically, with apex terminated by dense row of regular, thin, spiniform setae; tibial spur formula 0-1-1. Tarsi 5-5-4, all tarsomeres cylindrical, slightly tapered basally, truncate apically, progressively reducing in diameter; integument and setae similar to tibiae; protarsus longer than protibia. Protarsomere I slightly shorter than combined length of protarsomeres II and III, protarsomere IV slightly shorter than protarsomere V; relative ratios of mesotarsomeres similar except mesotarsomere I subequal to combined length of mesotarsomeres II and III; ratios of metatarsomeres similar. Pretarsal claws bifid, apical ramus sickle-shaped, inner ramus broadly rounded apically.

Elytra elongate, completely covering abdomen, surface imbricate; elytron basal width 1.08 mm, length 6.83 mm; each elytron with four indistinct costae; lateral margins parallel-sided, lateral margin comparatively straight until tapering inward in apical third, medial margin nearly straight until rounding at apex (Fig. 2); apex weakly acuminate.

Abdomen weakly imbricate, with scattered semi-decumbent to semi-erect setae.

♀: Latet.

###### Holotype.

♂, [Mexico:] F. Sumichrast [Francis E. Sumichrast (1828–1882), a famous Mexican collector who supplied biological specimens to many researchers and institutions during the 19^th^ Century] / Isth. [Isthmus] of Tehuantepec // F.C. Bowditch / coll. [Frederick Channing Bowditch (1854–1925) Collection, a wealthy amateur collector of Coleoptera] (MCZ). Unfortunately, the label data are no more specific than referencing the entire isthmus, which encompasses at its narrowest some 124 miles and varied terrain and habitats (e.g., centrally the Selva Zoque, a famous tropical forest region, ranging to dense jungle swamps in the North). It is therefore unclear as to precisely what environment in which to expect the present species. The specimen was likely collected during the same period in which Sumichrast collected birds from Tehuantepec for the United States National Museum (1868–1871) (Lawrence 1875).

###### Etymology.

The specific epithet honors Francis E. Sumichrast (1828–1882), collector of the holotype and many other fascinating species from southern Mexico during the mid-19^th^ Century.

##### Genus Clinops

Taxon classificationAnimaliaColeopteraRipiphoridae

Gerstaecker

Clinops Gerstaecker, 1855a: 16. Type species: Clinopsbadius Gerstaecker, 1855, by monotypy.

###### Diagnosis.

Body slender; elytra 3.0–3.4× as long as pronotal disc; coloration light to dark brown, with fine, short golden to light or dark brown setae; head with postocular genae expanded into lobes; compound eye not expanded beyond mandibular base and with a small extra-antennal sclerotous emargination; antenna with eleven antennomeres; male antenna with antennomeres I–III simple, IV–X with inner-facing, flabellate, compressed rami, XI similar in shape to preceding rami; female antenna similar to male with much shorter, pectinate, compressed rami; ultimate maxillary palpomere trapezoidal, apical width slightly less than maximum length, with blunt, truncate apex, not grossly enlarged; distal sensory duct on ultimate maxillary palpomere elongate, strongly oblique. Lateral aspect of pronotum with a ventrally bowed sulcus; pronotal disc without longitudinal medial impression; mesosternum weakly convex, without medial keel; metepisternum without elytron-receiving carina; posterior aspect of metepimeron slightly expanded. Metacoxa with strongly developed posterior flange; ventral surface of pro- and mesofemora in males without densely setose patch; tibial spur formula 0-0-2; pretarsal claws apically bifid, with or without a small, peg-like subsidiary tooth at midlength. Male genitalia with parameres weakly curved with apices widely separated from each other.

###### Comments.

The identity of *Clinops* has presented quite a historical challenge. Gerstaecker (1855a, 1855b) described the tibial spur formula for *Clinopsbadius* Gerstaecker as 0-?-2 (“*tibiis anticis muticis, mediis–?, posticis bispinosis*”), as both midlegs were missing in his female holotype (Figs 15–16). Falin (2003) proposed a formula of 0-0-2 for the genus, basing his conclusion on a specimen he interpreted as *C.badius* from TMSA (herein recognized as a separate species, *vide C.inexpectatus* sp. nov., *infra*). This interpretation for the genus is followed herein as it is consistent with what little is known of the tibial spur formulas for the three South African species we recognize: *C.badius* 0-?-2, *C.inexpectatus* 0-0-2, and *C.perpessus* sp. nov. 0-0-?. Accordingly, the tibial spur formula for *Clinops* differs from that of *Scotoscopus* Brenske and Reitter (*vide infra*), and the two genera are considered distinct, pending phylogenetic work throughout the subfamily.

There is a possibility that the differences observed between *C.inexpectatus* and *C.perpessus* are only sex differences rather than species distinctions. There are sexual dimorphisms known among pelecotomines, such as differences in the ultimate maxillary palpomeres of *Ancholaemus* Gerstaecker or color of the pronotal disc in *Scotoscopus*. Nonetheless, we believe the differences in head and pronotal shape reflect features specific to species, particularly as these are not known to be sexually variable in any other pelecotomines. Accordingly, we believe that the material described here represents distinct taxa. Naturally, the discovery of further material from a variety of localities will allow for further testing of this hypothesis.

It is interesting to note that while the form of the pretarsal claws has historically been used as a distinguishing feature for many genera, such as the conditions of bifid or pectinate, and in many cases such a difference does concord with other attributes, there is variation within *Clinops*. Among the species included here are those with strictly bifid claws, i.e., with a subapical ramus (tooth) that opposes the apical terminus of the claw, as well as one (*C.inexpectatus*) that has the typical bifid form coupled with the presence of a smaller, subsidiary tooth at about midlength (Fig. 26). It is therefore fascinating that with the addition of more and more such subsidiary teeth one progresses naturally into a pectinate condition. The claw of *Scotoscopus* (*vide infra*: Fig. 29) is somewhat similar albeit less pronounced, in that there is at least one, exceptionally short and blunt projection (appearing like a worn tooth) proximal to the initial ramus forming the bifid claw. It will be illuminating to more fully explore the complete ranges of variation in claw structure across the subfamily once more material and more species are discovered, and to develop clear homologies for the various elements in both bifid and pectinate claws and see how these various homologous elements distribute in a cladistic framework.

###### Distribution.

The genus is presently recorded only from South Africa. The precise locality from which Gerstaecker’s holotype of *C.badius* was collected is not known. Gerstaecker (1855a, 1855b) indicated the type locality only as “Caffraria”. This name (properly Kaffraria) was a historical, descriptive term for the southeastern region of the Eastern Cape (in which case the type locality was somewhere more southward coastally from the localities where the other two species were found). The newly described species, *C.inexpectatus* and *C.perpessus*, were collected on the eastern coast of South Africa, the former northeast of Durban toward Swaziland, and the latter somewhere in the region of Durban. Franciscolo (1952: antenna in his fig. 37) reported a female of “*Pelecotoma* sp.” from Cape Town, undoubtedly a misidentification for a specimen of *Clinops* (Batelka 2005), but the whereabouts of this specimen is unknown to us. The genus is probably widely distributed in woodlands of South Africa, perhaps escaping the attention of entomologists owing to its parasitoid biology.

##### Clinops perpessus sp. nov.

Taxon classificationAnimaliaColeopteraRipiphoridae

http://zoobank.org/B454A4B5-CCC7-4E42-AA5E-3DED1DD0FA53

Figs 4–5 , 6, 7 , 8–11 , 12–14

Pelecotominae new genus 2 gen. nov.: Falin 2003: 186.

###### Diagnosis.

**Figures 6, 7.**
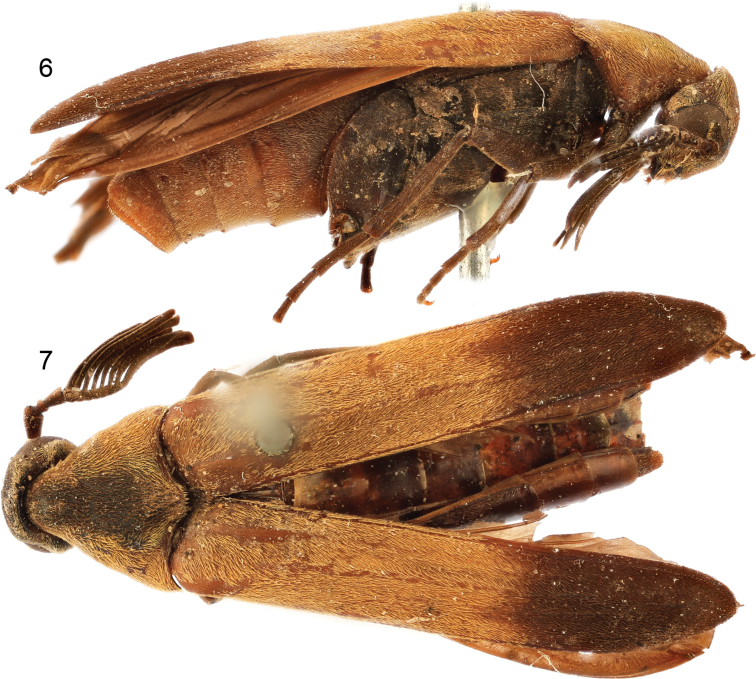
*Clinopsperpessus* sp. nov., holotype male. **6** lateral habitus **7** dorsal habitus.

Differs from *C.inexpectatus* by the only slightly elevated vertex above the pronotum (greatly elevated in *C.inexpectatus*: cf. Figs 6, 7 vs. Figs 19, 23), more robust head dorso-ventrally (1.63 mm vs. 1.08 mm in *C.inexpectatus* in which the head appears more flattened), elytral coloration (notably lighter basal two thirds in *C.perpessus*: cf. Figs 6, 7 vs. Figs 19, 20), by the absence of a mediolongitudinal shallow impression (furrow) in basal third of pronotal disc (present in *C.inexpectatus*: cf. Fig. 7 vs. Fig. 20), by the absence of a medioapical emargination to the median lobe of the pronotal posterior border (emargination present in *C.inexpectatus*), by base of pronotal disc wider than the length of the pronotal disc (pronotal disc as wide as long in *C.inexpectatus*), and by the stubbier terminal maxillary palpomere (cf. Fig. 5 vs. Fig. 22). Overall, the species is more similar to *C.badius*, as both species have the scarcely elevated vertex relative to the pronotum (e.g., Figs 6–7, 15–16). *Clinopsperpessus* differs from *C.badius* most notably in the shape of the terminal maxillary palpomere (cf. Fig. 5 vs. Fig. 18), the apically darkened elytra (uniformly colored in *C.badius*: cf. Figs 6–7 vs. Figs 15, 16), the more elongate antennal rami (cf. Fig. 4 vs. Fig. 17), and absence of a medioapical emargination to the median lobe of the pronotal posterior border (emargination present in *C.badius*).

**Figures 8–11.**
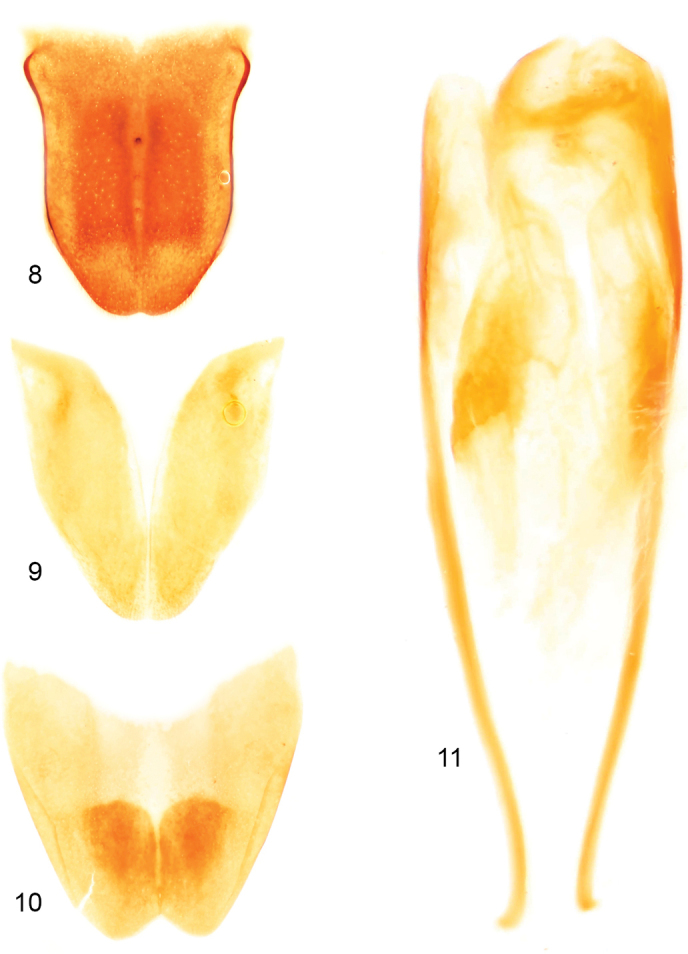
Male terminalia of holotype of *Clinopsperpessus* sp. nov. **8** tergum VII **9** tergum VIII **10** sternum VIII **11** sternum IX.

**Figures 12–14.**
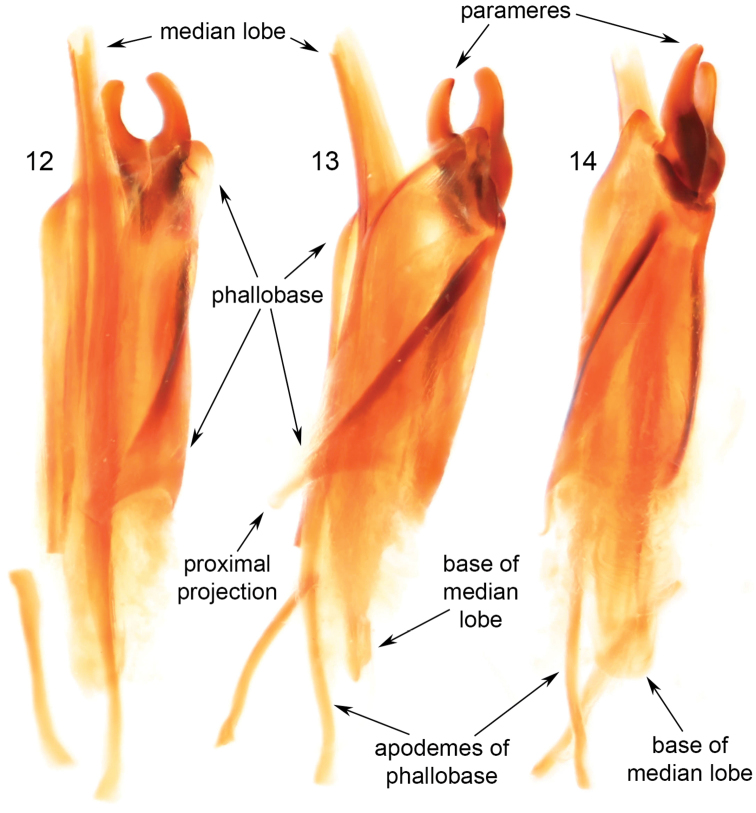
Male genitalia of holotype of *Clinopsperpessus* sp. nov. (tegmen = phallobase + parameres / parameres = lateral lobes, gonoforceps / median lobe = aedeagues). **12** dorsal oblique view **13** lateral oblique view **14** ventral oblique view.

###### Description.

♂: General size and appearance typical of Pelecotominae. Size 9.75 mm from tip of abdomen to base of antennae, 2.54 mm wide at base of pronotum. Body largely dark brown, slightly lighter reddish brown on lateral thirds of pronotum, basal two thirds of elytra, and apical abdominal sterna (Figs 6–7).

Head ovoid, approximately 1.02× longer than wide in facial view, medial length 1.67 mm, maximum width (across compound eyes) 1.63 mm. Vertex convex dorsally and posteriorly, as wide as lower face (below compound eyes), rising high above compound eyes in facial view, sloping uniformly to meet and slightly overlap pronotal anterior margin, with weak medially impressed line, disappearing posteriorly. Dorsal, lateral, and facial aspects of head with fine, decumbent, golden to fuscous setae, particularly numerous on face between compound eyes and vertex, abundant on genae; integument dull, with minute, nearly contiguous punctures separated by apparently smooth to imbricate integument. Compound eye of moderate size, encompassing much of medial third of lateral surface of head, finely faceted, emarginate in upper third (emargination deeper than in *Z.sumichrasti*, such that compound eye nearly appears bisected in facial view: cf. Figs 3 vs. 4). Postocular gena expanded into lobe. Frons broad, with antennal torulus laterally directed, antennal toruli separated by distance equal to length of scape, compound eyes separated by distance slightly less than maximum compound eye length. Malar space elongate, more than one-half length of scape, slightly less than basal mandibular width. Mandible short, slightly curved, with short, acute subapical tooth. Maxillary palpus long, tetramerous, terminal palpomere largest, trapezoidal, its apical width slightly less than maximum length, with blunt, truncate apex, not flattened or grossly enlarged (greatly enlarged and flattened in *Ancholaemus* Gerstaecker and *Micholaemus* Viana), distal sensory duct elongate, strongly oblique.

Antenna consisting of eleven antennomeres; antennomere I longer than wide, slightly curved to approximate curvature of compound eye; antennomere II short, slightly wider than long; antennomere III longer than antennomere II, length approximately 1.3× apical width, apical margin truncate. Antennomeres IV–XI greatly dissimilar from preceding antennomeres; antennomeres IV–X with internally facing, compressed rami; bases of antennomeres IV–X short and of similar lengths; rami IX and X elongate, extending to apex of antennomere XI; remaining rami progressively shorter from X to IV. Antennomere XI expanded, similar in shape to rami of preceding antennomeres. Total length of antennomere XI approximately 2× length of bases of antennomeres IV–X combined.

Pronotum with semi-decumbent to decumbent, fine, golden setae except in medial third such setae fuscous, integument dull, and weakly and contiguously punctate, with punctures more indistinct anteriorly and posteriorly, integument becoming imbricate. Pronotum triangular in shape, narrowed anteriorly; anterior margin broadly rounded; posterior margin sinuate and generally trilobed, with medial lobe scarcely broader than mesoscutellum and rounded (not emarginate: distinctly emarginate in *C.inexpectatus*, *vide infra*); lateral margins generally straight, converging anteriorly, convex ventrally to propleurae; propleuron well developed. Pronotal disc wider at base than length, without mediolongitudinal carina or impression; lateral aspect with a ventrally bowed sulcus. Mesonotum obscured by elytra. Mesoscutellum (mesoscutellar shield) short, flat, parallel-sided, with broadly rounded apex; integumental sculpturing and setation as on pronotal disc. Metanotum obscured by elytra.

Lateral and ventral aspects of pterothorax typical of subfamily. Mesepisternum weakly and faintly imbricate, with scattered minute punctures, fused with mesosternum, with scattered decumbent setae. Mesepimeron forming prominent, rectangular sclerite separated from mesepisternum by deep sulcus; sculptured and setation as on mesepisternum. Metepisternum an elongate, narrow rectangular sclerite, with sculpturing and setation as on mesepisternum; metasternum massive, weakly imbricate and with decumbent setae more numerous than on metepisternum. Metepimeron slightly expanded posteriorly, extending anteriorly to wing base as narrow (slightly more narrow than metepisternum), sclerotized band; weakly imbricate with scattered setae.

Legs typical for subfamily; coxae, trochanters, and femora weakly, irregularly, almost indistinctly punctate on otherwise smooth integument with decumbent, golden to lightly fuscous setae; metacoxa with strongly developed posterior flange; femora without densely setose patches ventrally; tibiae straight, cylindrical, broadened slightly apically, with apex terminated by dense row of regular, thin, spiniform setae; tibial spur formula 0-0-? (hind legs missing in holotype). Tarsi 5-5-[4, metatarsus presumed to have had four tarsomeres], all tarsomeres cylindrical, very slightly tapered basally, truncate apically; integument and setae similar to tibiae; protarsus longer than protibia. Protarsomere I subequal to combined length of protarsomeres II and III, protarsomere IV less than one-half length protarsomere V; relative ratios of basal mesotarsomeres similar (apical tarsomeres of meso- and metatarsi missing in holotype). Pretarsal claws bifid, apical and inner rami both sickle-shaped and acutely pointed, without any midlength or subsidiary teeth.

Elytra elongate, completely covering abdomen, surface imbricate with minute, weak, nearly contiguous punctures; elytron basal width 1.27 mm, length 8.21 mm; each elytron with four indistinct costae; lateral margins parallel-sided, lateral margin comparatively straight until tapering inward in apical fifth, medial margin nearly straight until rounding at apex; apex weakly acuminate.

Abdomen with terga weakly and faintly imbricate; sterna imbricate with scattered minute punctures, with scattered decumbent, fine setae; male terminalia as depicted in figures 8–14.

♀: *Latet*.

###### Holotype.

♂, [South Africa: KwaZulu-Natal: eThekwini:] Port / Natal / 49 29 [on underside of label] [no collector or date] (NHMUK). The “49 29” on the underside of the label corresponds to the 29^th^ accession of 1849 (M. Barclay, pers. comm.). This accession was a collection of 1627 insects, including 965 Coleoptera, from Port Natal, South Africa collected by Wilhelm Gueinzius (1813–1874), and sold to the Natural History Museum through Samuel Stevens’ (1817–1899) auctions at 24 Bloomsbury Street in London during December 1849 (M. Barclay, pers. comm.). Gueinzius, a German naturalist who spent most of his life in present-day South Africa, lived in the area of Port Natal (settled along the Tugela River) from 1841 until late in 1843 when he returned to Cape Town after British troops looted his home during conflict with the Zulus and Boers. He returned to the Natal area in mid-1844 and remained there until shortly before his death. Since the present specimen was auctioned and accessioned in December 1849 it can safely be presumed it was collected sometime between 1844 and this date. A handwritten label from the 19^th^ Century accompanying the specimen reads “closely resembles the figure of *Ancholaemuslyciformis*, but that is from Brazil.” The handwriting generally matches that of George C. Champion (1851–1927). The specimen’s terminalia were apparently dissected by the late John K. Bouseman (1936–2006) (the sclerites are stored within a genitalia vial along with the specimen), who labeled the specimen “Rhipiphoridae [sic] gen. et sp. nov. ♂ Det. Bouseman ’71 [1971]”.

###### Etymology.

The specific epithet is taken from the Latin, meaning “suffer with patience” or “endure”, and is a reference to the vast time over which this species has awaited description.

##### Clinops inexpectatus sp. nov.

Taxon classificationAnimaliaColeopteraRipiphoridae

http://zoobank.org/73E8653E-4466-4A01-8466-B140D7B2DA59

Figs 19–22 , 23–26

“Clinopsbadius Gerstaecker”: Falin 2003: 175, 439 [misidentification].

###### Diagnosis.

Refer to diagnosis of *C.perpessus* (*vide supra*).

###### Description.

♀: General size and appearance typical of Pelecotominae. Size 10.02 mm from tip of elytra to mandibles, 2.14 mm wide at base of pronotum. Body largely dark brown, slightly lighter reddish brown on humeral parts of elytra, and apical abdominal sterna (Figs 19, 20).

Head hexagonal from facial view, approximately 1.25× longer than wide in facial view, medial length 1.80 mm, maximum width (across compound eyes) 1.08 mm. Vertex convex dorsally and posteriorly, as wide as lower face (below compound eyes), rising high above compound eyes in facial view, sloping uniformly to meet and distinctly overlap pronotal anterior margin. Dorsal, lateral, and facial aspects of head with fine, sparse, golden setae, particularly numerous on face between compound eyes, abundant on genae; integument dull, with deep, nearly contiguous punctures separated by smooth integument. Compound eye of moderate size, length 0.84 mm, width 0.29 mm, encompassing much of medial third of lateral surface of head, finely faceted, emarginate in upper third. Postocular gena expanded into lobe. Frons broad, with antennal torulus laterally directed, antennal toruli separated by distance equal to length of scape, compound eyes separated by distance slightly less than maximum compound eye length. Malar space elongate, more than one-half length of scape, slightly less than basal mandibular width. Mandible short, slightly curved, with short, acute subapical tooth. Maxillary palpus long, tetramerous, apical palpomere largest, trapezoidal, its apical width slightly less than maximum length, with blunt, truncate apex, distal sensory duct elongate, strongly oblique.

**Figures 15–18.**
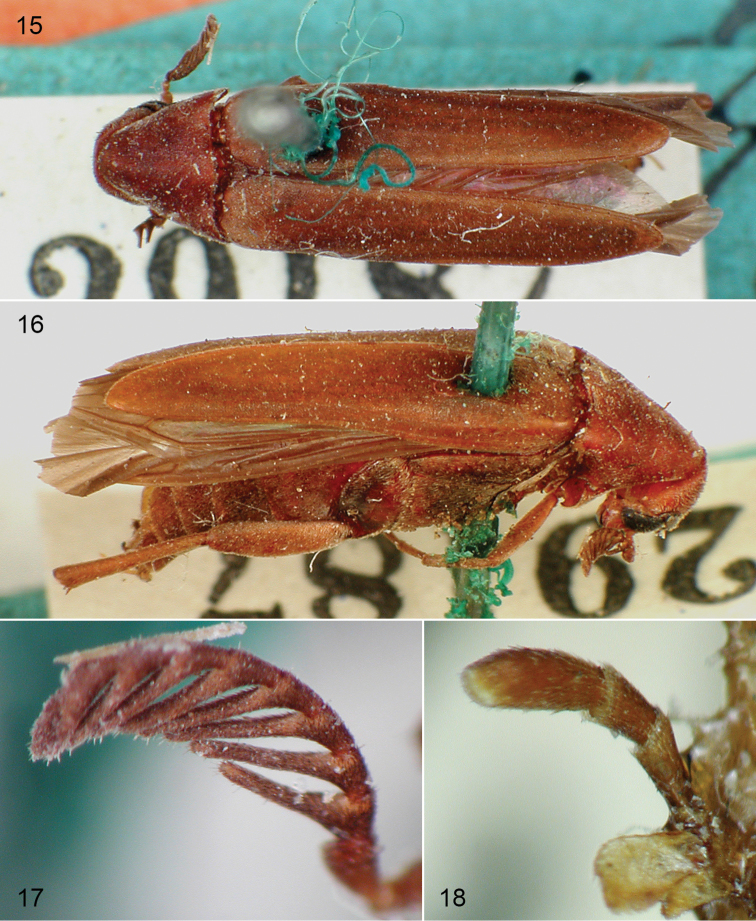
*Clinopsbadius* Gerstaecker, holotype female. **15** dorsal habitus **16** oblique lateral habitus **17** detail of right antenna **18** maxillary palpus (dissected and mounted by Gerstaecker).

**Figures 19–22.**
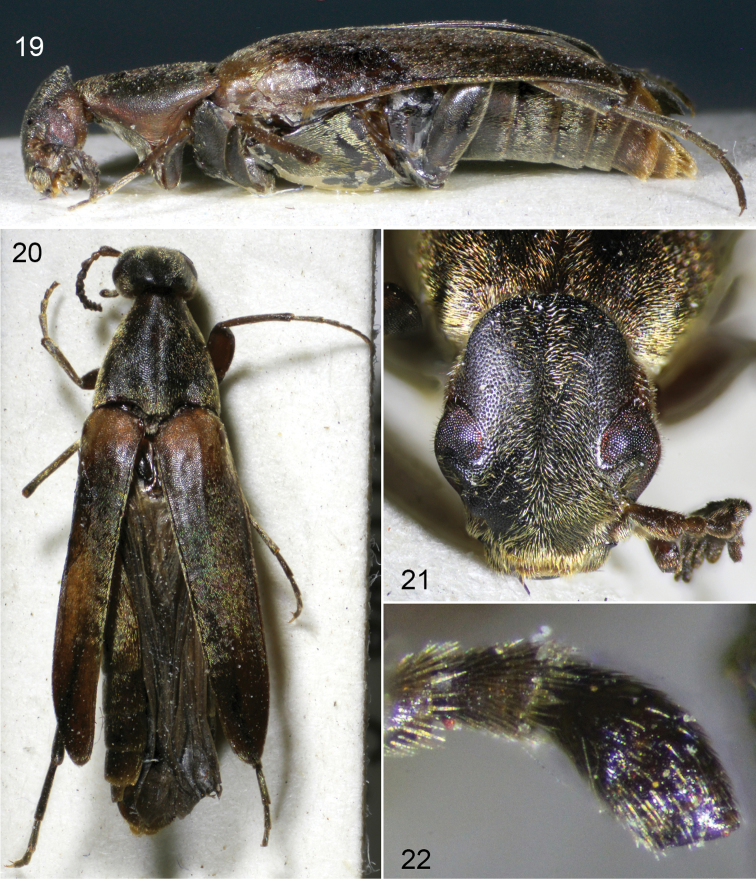
*Clinopsinexpectatus* sp. nov., holotype female. **19** lateral habitus **20** dorsal habitus **21** facial view **22** maxillary palpus.

Only left antenna without apical antennomere preserved, antenna consisting probably of eleven (10 preserved) antennomeres; antennomere I longer than wide, slightly curved to approximate curvature of compound eye; antennomere II short, distinctly longer than wide, much narrowed in basal third; antennomere III longer and wider than antennomere II, length approximately 1.5× apical width, apical margin as in antennomere II; antennomeres IV–X greatly dissimilar from preceding antennomeres, with internally facing, compressed rami truncated apically, bases short and of similar lengths; rami elongate, about 2.0× as long as their respective base.

Pronotum with fine, golden setae except in medial third such setae fuscous, integument dull, and weakly and contiguously punctate, with punctures more indistinct anteriorly and posteriorly, integument becoming imbricate. Pronotum triangular in shape, narrowed anteriorly, median length 2.30 mm; anterior margin broadly rounded; posterior margin sinuate and generally trilobed, with medial lobe broader than mesoscutellum and distinctly emarginate (rounded in *C.perpessus*); lateral margins generally straight, converging anteriorly, convex ventrally to propleurae; propleuron well developed. Pronotal disc as wide as long, with mediolongitudinal shallow impression in basal third; lateral aspect with a ventrally bowed sulcus. Mesonotum obscured by elytra. Mesoscutellum short, mesoscutellar shield with deep medial furrow, parallel-sided, with broadly rounded apex; integumental sculpturing and setation as on pronotal disc. Metanotum obscured by elytra.

Lateral and ventral aspects of pterothorax typical of subfamily. Mesepisternum weakly and faintly imbricate, with scattered minute punctures, fused with mesosternum, with scattered decumbent setae. Mesepimeron forming prominent, rectangular sclerite separated from mesepisternum by deep sulcus; sculptured and setation as on mesepisternum. Metepisternum an elongate, narrow rectangular sclerite, with sculpturing and setation as on mesepisternum; metasternum massive, weakly imbricate and with decumbent setae more numerous than on metepisternum. Metepimeron slightly expanded posteriorly, extending anteriorly to wing base as narrow (slightly more narrow than metepisternum), sclerotized band; weakly imbricate with scattered setae.

Legs typical for subfamily (left front and mid-leg incomplete); coxae, trochanters, and femora weakly, irregularly, almost indistinctly punctate on otherwise smooth integument with decumbent, golden setae; metacoxa with strongly developed posterior flange; femora without densely setose patches ventrally; tibiae straight, cylindrical, broadened slightly apically, with apex terminated by dense row of regular, thin, spiniform setae; tibial spur formula 0-0-2, metatibial spurs well visible. Tarsal formula 5-5-4, all tarsomeres cylindrical, very slightly tapered basally, truncate apically; integument and setae similar to tibiae; protarsus longer than protibia. Protarsomere I subequal to combined length of protarsomeres II and III, protarsomere IV less than one-half length protarsomere V; relative ratios of basal mesotarsomeres similar. Pretarsal claws with apical and inner teeth both sickle-shaped and acutely pointed, with small, peg-like subsidiary tooth at midlength (Fig. 26).

Elytra elongate, completely covering abdomen, surface shining with minute, weak, nearly contiguous punctures; elytron basal width 1.15 mm, length 6.10 mm; without costae; lateral margins parallel-sided, lateral margin comparatively straight until tapering inward in apical fifth, medial margin nearly straight until rounding at apex; apex weakly acuminate.

Abdomen with terga weakly and faintly imbricate; sterna imbricate with scattered minute punctures, with scattered decumbent, fine setae, ovipositor shallowly protruded.

♂: Unknown.

###### Holotype.

♀, S[outh]. Afr[ica].: Zululand / Hluhluwe Game Res. / 28.05. S -32.04 E // 20.11.1992 [20 November 1992]; E.-Y: 2839 / fruittraps, woodysav [?] / leg. Endrödy - Younga // Clinops / badius / Gerstaecker 1855 / Det. ZH Falin [20]’09”.

###### Etymology.

The specific epithet is taken from Latin, meaning “unexpected”, and refers to the surprise that it was undescribed upon re-examination by JB.

**Note.** Although the identification label is dated 2009, the specimen was earlier identified and used by ZHF as *C.badius* in Falin (2003).

**Figures 23–26.**
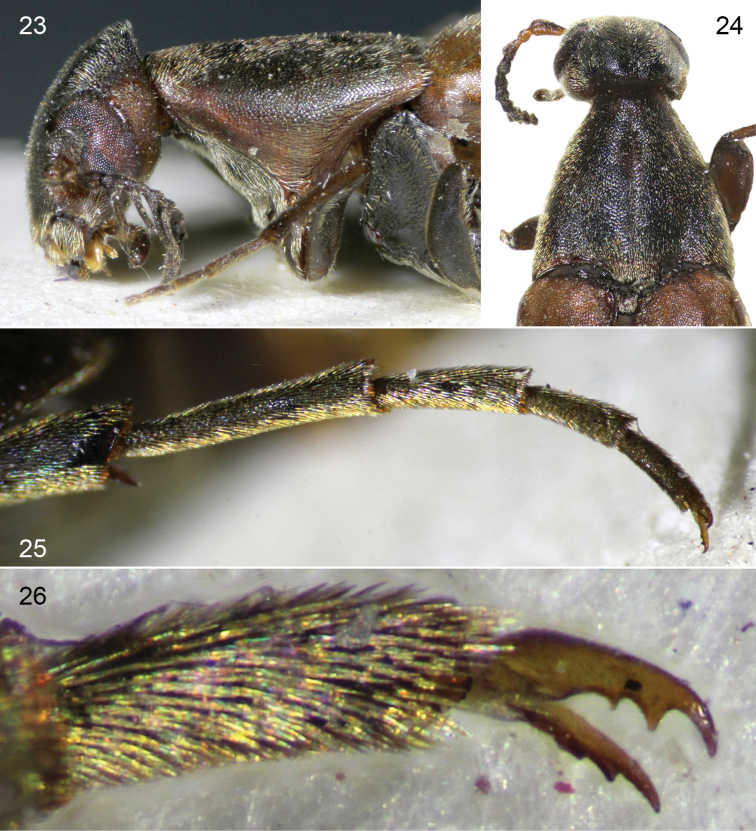
*Clinopsinexpectatus* sp. nov., holotype female. **23** lateral detail of head and pronotum **24** dorsal detail of head and pronotum **25** metatarsus **26** detail of metapretarsal claws.

**Figures 27–29.**
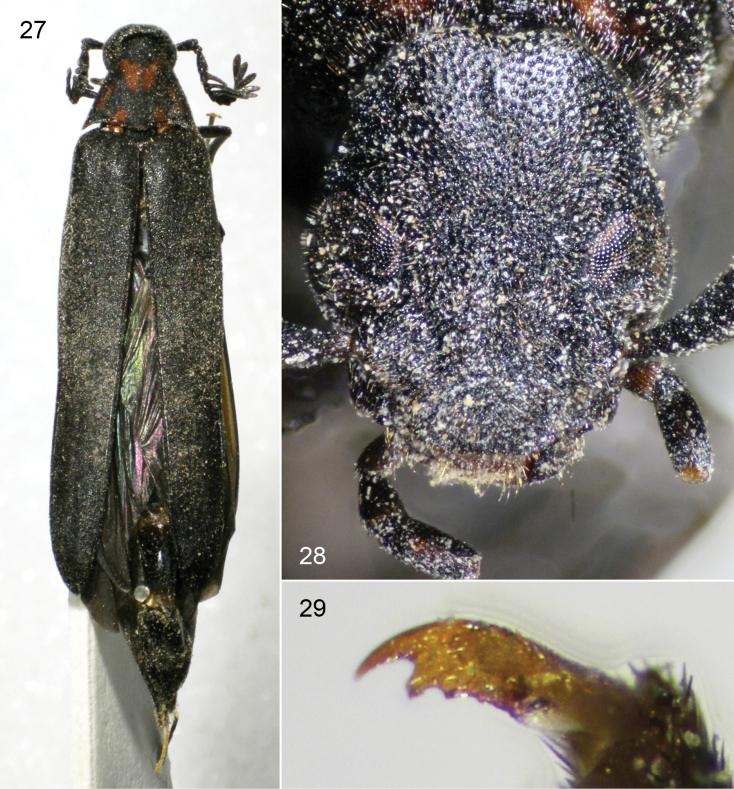
*Scotoscopusspectabilis* (Schaufuss), female from Crete. **27** dorsal habitus **28** facial view **29** detail of metapretarsal claws.

##### Genus Scotoscopus

Taxon classificationAnimaliaColeopteraRipiphoridae

Brenske & Reitter, resurrected status

Scotoscopus Brenske & Reitter, 1884: 92. Type species: Scotoscopuscarbonarius Reitter in Brenske & Reitter, 1884 (= Clinopsspectabilis Schaufuss, 1872), by monotypy.

###### Diagnosis.

Body slender; elytra 4.0–4.8× as long as pronotal disc, coloration of head, elytra, meso- and metathorax and abdomen dark brown or black, pronotum bright red in males and dark-red with black markings, setae dark, sparsely distributed and indistinct; head with postocular genae expanded into lobes; compound eye not expanded beyond mandibular base and with a small extra-antennal sclerotous emargination; antenna with eleven antennomeres; male antenna with antennomeres I–III simple, III compressed, almost lenticular, IV–X with inner-facing, flabellate, compressed rami, XI similar in shape to preceding rami; female antenna similar to male, antennomere III long and cylindrical, rami much shorter, pectinate, compressed; ultimate maxillary palpomere cylindrical, apical width 3× less than maximum length, with blunt, truncate apex, not grossly enlarged; distal sensory duct on ultimate maxillary palpomere elongate, strongly oblique. Lateral aspect of pronotum with a ventrally bowed sulcus; pronotal disc without longitudinal medial impression; mesosternum weakly convex, without medial keel; metepisternum without elytron-receiving carina; posterior aspect of metepimeron slightly expanded. Metacoxa with strongly developed posterior flange; ventral surface of pro- and mesofemora in males without densely setose patch; tibial spur formula 0-2-2; pretarsal claws bifid, with blunt, small subsidiary tooth proximal to inner bifid ramus (Fig. 29). Male genitalia with parameres strongly curved, with apexes overlapping each other.

###### Comments.

Batelka (2005) recognized that the type species of the genus was a junior synonym of *Clinopsspectabilis* Schaufuss, and he accordingly synonymized the two genera. However, based on the differences in tibial spur formula between *Clinops* (*vide supra*) and the type species of *Scotoscopus*, we herein reinstate the genus.

##### Scotoscopus spectabilis

Taxon classificationAnimaliaColeopteraRipiphoridae

(Schaufuss) comb. nov.

**Figures 30, 31.**
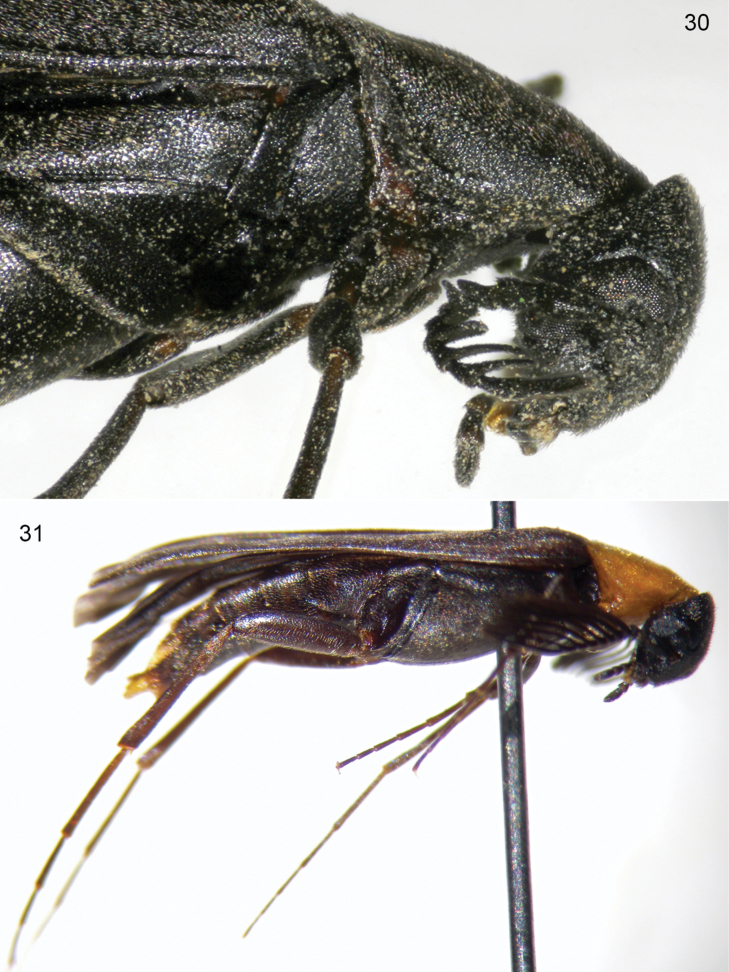
*Scotoscopusspectabilis* (Schaufuss). **30** lateral detail of head and pronotum (female from Crete) **31** lateral habitus (male from Peloponnese, photograph courtesy of M. Fikáček).

**Figures 32, 33.**
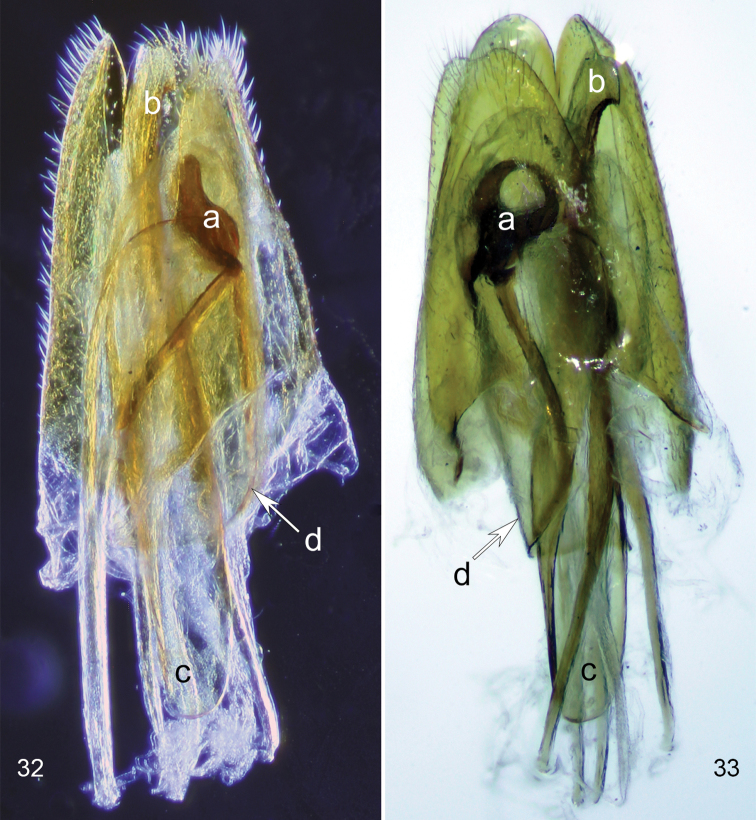
*Scotoscopusspectabilis* (Schaufuss), male terminalia (specimen from Crete). **32** lateral, dark-field view **33** dorsal-ventral view. a) parameres, b) apical hook of median lobe, c) base of median lobe, d) phallobase.

Figs 27–29 , 30, 31 , 32–33

Clinops spectabilis Schaufuss, 1872: 276.
Scotoscopus carbonarius Reitter in Brenske & Reitter, 1884: 93. Synonymy *vide*Batelka (2005).

###### Diagnosis.

As for the genus (*vide supra*).

###### Material examined.

1♂, Greece: Pelopónnisos / Taïyetos Mts., 950 – / 1800 m, 15.–19.v.1990 [15–19 May 1990] / Zool. Mus. Copenh. Exp. [p] // Scotoscopus / carbonarius Rtt. / det. C. Wurst 99 [handwritten] (ZMUC).

###### Distribution.

Hitherto, the species was known from the holotypes of the two synonymous taxa collected in Turkey (Antakya) and Mount Parnassus (central Greece) and subsequently from approximately a dozen specimens from Crete (Cretan Archipelago, Greece) (Batelka 2005, 2007). This is the first record of the species from the Peloponnese (southern Greece).

## Discussion

The study of Pelecotominae, like any rare group of organisms, is hampered by a dearth of information and available material. Several groups are known only from historical type material, one sex, and from little more than type localities. Beyond this, the biology, ecology, and immature stages of most species remains utterly unknown and modeling of ecological niches is presently impossible without a greater variety of collecting localities across diverse habitats. The closest one might come to having sufficient material upon which to base ecological models or explore intraspecific variation would be some of the South American species, such as *Ancholaemuslyciformis* Gerstaecker, where comparatively larger series of specimens are known from a broad range of localities. Otherwise, with such meagre material at hand for Pelecotominae, further exploration and surveys are desperately needed in order to significantly advance our knowledge of this lineage. In the interim, the systematics of the subfamily is improving by slow iterations, to which the present study is but one small step.

Among the many present challenges toward a systematization of the Pelecotominae is the clarification of traits for many species and genera, and whether or not these features are of broader phylogenetic significance. For example, the tibial spur formula has been used to distinguish genera across the subfamily. As shown herein by the revised diagnosis of *Clinops*, this feature alone has been difficult to interpret. For example, in many taxa the mesotibial spurs are exceedingly small and easily overlooked (particularly if certain specimen preparations obscure a direct view of the inner apical mesotibial articulation), or in older specimens may be damaged and missing, and thereby understandably miscoded as absent, ultimately leading to confusion in the placement of certain specimens and species. More critically, a comprehensive phylogeny of the subfamily is lacking and presently not possible and it therefore remains speculative as to whether this feature (or any of the traits used to recognize genera in the subfamily) will be shown to support clades at any rank, or whether they will prove to be rampantly homoplastic at anything above the level of species. Indeed, once a phylogeny is resolved for the subfamily, it could be discovered that placing an emphasis on tibial spur formula in the circumscription of genera is misguided and does not actually characterize natural groups. Furthermore, the forms of the maxillary palpi and pretarsal claws are more quantitative than qualitative and so likewise require testing in a cladistic framework. This is not to say that the tibial spur formula, pretarsal claw structure, or maxillary palpus form will not ultimately prove to be consistent in a phylogenetic framework and support clades traditionally recognized as genera, merely that in the absence of such a resolved topology their validity is equivocal. Moreover, additional character systems such as the male genitalia remain to be explored comparatively. This is understandable as males are not known for all taxa, but nonetheless represent one of any number of potentially valuable sources of characters. The male genitalia of Pelecotominae are not often figured and, where known, there is comparatively little variation in overall form (e.g., Rivnay 1929; Selander 1957). Nonetheless, a thorough documentation and comparative morphological exploration of pelecotomine genitalia would be worthwhile and may reveal at least a few characters of phylogenetic significance. The same is true for larvae, with few pelecotomine immature stages documented in the literature or in a comparative light (Lawrence et al. 2010). Thus, one of the most important future developments for the study of Pelecotominae is the extensive sampling of taxa in the field so as to build up sufficient material, sexes, and larvae to not only clarify the biology and ecology of these species, but to permit the resolution of relationships and character evolution across the clade.

## Supplementary Material

XML Treatment for Zapotecotoma

XML Treatment for Zapotecotoma sumichrasti

XML Treatment for Clinops

XML Treatment for Clinops perpessus

XML Treatment for Clinops inexpectatus

XML Treatment for Scotoscopus

XML Treatment for Scotoscopus spectabilis
